# Analytical Ultracentrifugation Detects Quaternary Rearrangements and Antibody-Induced Conformational Selection of the SARS-CoV-2 Spike Trimer

**DOI:** 10.3390/ijms241914875

**Published:** 2023-10-03

**Authors:** Giuditta Guerrini, Dora Mehn, Francesco Fumagalli, Sabrina Gioria, Mattia Pedotti, Luca Simonelli, Filippo Bianchini, Davide F. Robbiani, Luca Varani, Luigi Calzolai

**Affiliations:** 1 European Commission, Joint Research Centre (JRC), 21027 Ispra, Italy; giuditta.guerrini@ec.europa.eu (G.G.); dora.mehn@ec.europa.eu (D.M.); francesco-sirio.fumagalli@ec.europa.eu (F.F.); sabrina.gioria@ec.europa.eu (S.G.); 2 Institute for Research in Biomedicine, Università della Svizzera Italiana, 6500 Bellinzona, Switzerland; mattia.pedotti@irb.usi.ch (M.P.); luca.simonelli@irb.usi.ch (L.S.); filippo.bianchini@irb.usi.ch (F.B.); drobbiani@irb.usi.ch (D.F.R.)

**Keywords:** SARS-CoV-2, spike, trimer, antibody, AUC, analytical ultracentrifugation, sedimentation, conformation

## Abstract

Analytical ultracentrifugation (AUC) analysis shows that the SARS-CoV-2 trimeric Spike (S) protein adopts different quaternary conformations in solution. The relative abundance of the “open” and “close” conformations is temperature-dependent, and samples with different storage temperature history have different open/close distributions. Neutralizing antibodies (NAbs) targeting the S receptor binding domain (RBD) do not alter the conformer populations; by contrast, a NAb targeting a cryptic conformational epitope skews the Spike trimer toward an open conformation. The results highlight AUC, which is typically applied for molecular mass determination of biomolecules as a powerful tool for detecting functionally relevant quaternary protein conformations.

## 1. Introduction 

The proper assembly of multimeric proteins with quaternary structure and large conformational rearrangements involving domains or monomers are crucial for their function. Full atomistic structure determination is often the only reliable way to access such information, although it is challenging in the frequent cases when molecules undergo transient molecular rearrangements. Even computational methods fail to accurately predict large domain movements, despite their progress in secondary and tertiary structure prediction [1].

Developed at the beginning of the last century [2], analytical ultracentrifugation (AUC) is mainly seen as a tool complementary to size exclusion chromatography that is used to obtain information about quantity and size of macromolecules [3,4,5]. It separates molecules based on their sedimentation velocity when centrifuged at high speed in solution, without demanding sample preparation. The resulting sedimentation coefficient distributions’, expressed in Svedberg (S), are well reproducible within and between laboratories [6,7]. In addition to mass, the sedimentation speed of molecules is also affected by their shape. “Deviation” from the spherical shape leads to larger friction during sedimentation and results in lower sedimentation coefficient. Theoretical estimation of sedimentation speed, including models for different molecular shapes, can be used to match the experimental AUC data to three-dimensional molecular structures [4,8,9,10], with good agreement between measured and calculated sedimentation coefficient values for various oligomeric proteins [11,12] and other molecules. Given the effect of shape on the sedimentation velocity, we here employ AUC to probe molecular structural heterogeneity of the SARS-CoV-2 Spike protein.

Coronavirus entry into the host cell is mediated by the glycosylated Spike protein [13], which is a homotrimer embedded in the viral envelope. Each monomer consists of an ectodomain, a transmembrane domain and an intracellular tail. The ectodomain spans between two subunits, S1 and S2; the former is responsible for binding to the host cell receptor ACE2, and the latter—after cleavage and large conformational rearrangements—for fusion of the virus envelope with the host cell membrane [13,14,15].

The S1 subunit contains an N-terminal domain (NTD), a receptor binding domain (RBD), and subdomains 1 and 2 (SD1 and SD2). The RBD is the primary target for antibodies capable of neutralizing the virus, generated by the natural immune response after infection/vaccination or developed for monoclonal antibody-based immunotherapy, a proven and safe life-saving treatment for at risk COVID-19 patients [16].

The Spike protein domains have been hinted, and at times proven [17], to undergo conformational rearrangements. The RBD, for instance, was shown to stably adopt either an “up” or “down” configuration, with only the former capable of engaging ACE2 and lead to infection [18,19]. Long-range allosteric effects play important and not yet fully understood role in the distribution of Spike molecular conformers. For example, the first stable SARS-CoV-2 variant, which became dominant mere weeks after the virus breakout in Europe in 2020, involved substitution of a single residue in the S1 domain (D614G). This mutant was shown to favour an RBD up conformation [20] despite the RBD being 40 Angstrom away and seemingly not connected, resulting in easier engagement of ACE2 and consequent higher infectivity of this viral variant [20,21].

It is reasonable to expect that conformational movements or plasticity of other Spike domains exist, involving transient—if not stable—configurations. Indeed, other viral surface proteins [22,23] are frequently suggested to undergo large conformational movements, either at the single molecule level or in the context of the entire viral surface (sometime referred to as ‘breathing’) [24,25]. Direct, experimental observation of these conformational states is difficult at best, impossible at worst.

Beyond the above, many viral proteins exist in a particular conformation on the virus surface, but undergo extensive conformational changes to achieve fusion with the host cell membranes and consequently infectivity. This is required, for instance, because the so-called fusion peptide, a protein sequence often responsible for initiating viral fusion, must be initially inaccessible (fusion outside the target cell can lead to virus inactivation) and only exposed in close proximity of the host target cell membrane or inside it. Conformational rearrangements from so-called ‘pre- to post-fusion’ states can be very extensive, including switch from dimeric to trimeric conformations (e.g., Flaviviruses), cleavage of protein fragments and separation of subunits [26]. While stable pre- and post-fusion conformations have been structurally determined [27], observing the transient intermediate states between the these conformations is technically challenging and only seldom achieved. Characterizing these molecular movements is crucial not only to understand viruses and their function, but also to provide new therapeutic targets. Blocking the rearrangements required for viral fusion by antibodies or other means, can in fact result in virus neutralization.

Here we show that analytical ultracentrifugation can probe the presence of sub-populations of molecular conformers in the SARS-CoV-2 Spike protein, as well as evidence of conformational selection by neutralizing monoclonal antibody (NAb).

## 2. Results

**Figure 1:**
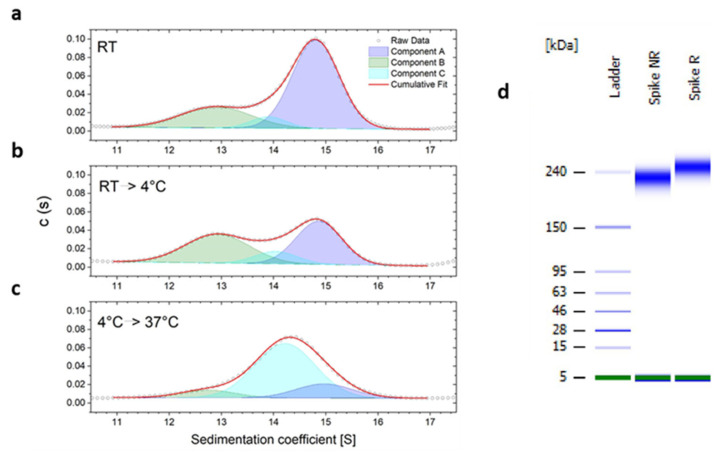
(**a**) AUC sedimentation coefficient distribution of D614G Spike protein measured at 20 °C after pre-incubation at a| room temperature, (**b**) 4 °C and (**c**) 4 °C then 37 °C. Results of deconvolution shown with various colours—Components A, B, C, (**d**) SDS-PAGE of the D614G Spike protein sample used for AUC under non-reducing (Spike NR) and reducing conditions (Spike R).

The sedimentation coefficient distribution of the D614G Spike variant ectodomain measured by AUC (after pre-incubation at room temperature for 10 min) shows a main peak around 14.8 S, corresponding to a calculated molecular weight (MW) of about 530 kDa, in line with the expected mass of the Spike trimer (Figure 1a). However, AUC also detects a peak at 12.9 S, corresponding to a calculated MW of about 425 kDa, not consistent with a Spike monomer or dimer. This cannot arise from a truncated Spike form, either, since SDS-PAGE analysis confirms the presence of a single species and no smaller fragments (Figure 1d). Deconvolution of the sedimentation coefficient distribution reveals the presence of a third peak (conformer sub-population) around 14.0 S.

A single molecular mass with different sedimentation coefficients can be explained by the simultaneous presence of spatial conformations with different frictional coefficients resulting in sedimentation velocity changes. When incubated at 4 °C (Figure 1b), the contribution of the 12.9 S component increases, while after subsequent incubation at 37 °C, the species at 14.8 S and 12.9 S become less populated, with corresponding increase of the 14.0 S component (Figure 1c). This is in line with the idea of coexistence of transient conformers, whose interchange and relative abundance change with temperature.

**Figure 2:**
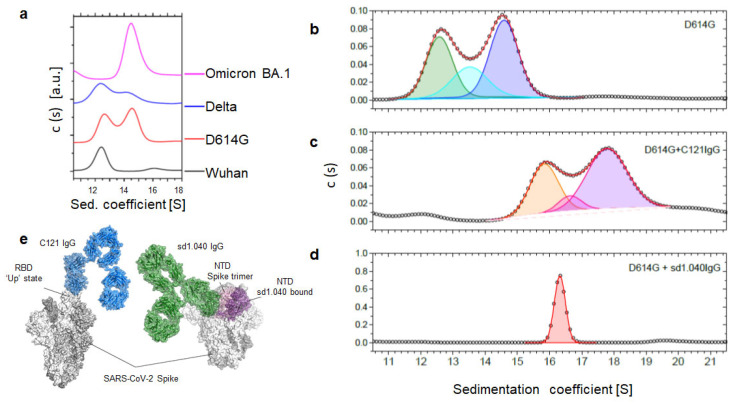
(**a**) AUC sedimentation coefficient distribution of the wild type Spike protein (Wuhan, black) and the major variants of concern: D614G (red), Delta (blue) and Omicron BA.1 (pink). (**b**–**d**) Effects of antibody binding on the different quaternary conformations of D614G Spike protein. (**b**): protein alone; (**c**): spike protein bound to C121 IgG; (**d**): spike protein bound to sd1.040 IgG. (**e**) Structures of Spike-C121Ab and Spike-sd1.040 complexes.

The distribution of peaks in AUC is altered not only by temperature, but also by the Spike protein sequence. Recombinant Spikes from other natural viral variants of concern (ancestral virus, D614G, Delta and Omicron BA.1) show different distribution of peaks in AUC, indicating the presence of different structural sub-populations that might affect the virus infectivity and recognition by neutralizing antibodies (Figure 2a).

AUC reveals that the Spike protein exists in an equilibrium of conformers with different sedimentation velocity and thus shape. The first thought is that they could relate to the RBD up/down conformations, which were shown to exist in experimentally determined structures [28,29].

According to calculations performed [10] on available PDB structures of the Spike with one or more RBDs in the up conformation, the different RBD position changes the predicted sedimentation coefficients by 0.1–0.2 S, which would not justify the observed experimental differences of ~1.9 S in AUC (Table 1). Cryo-TEM [30] and hydrogen/deuterium exchange experiments together with melting curves [30,31] also suggested the presence of other Spike quaternary conformations that cannot be explained by the relatively small rearrangement of one or more RBD domains in the up/down position. By contrast, S coefficients calculated on a manual structural model of the Spike trimer stretched into a conformation that opens up the trimer (Table 1, 7EB5MOD) are compatible with the experimentally observed values. Although such structure is fully artificial, it illustrates how previously uncharacterized open conformations could be responsible for the higher frictional forces resulting in lower S peaks in AUC.

In an effort to further characterize the Spike conformers, we performed AUC experiments in the presence of antibodies against it.

Upon addition of antibody C121, which binds to both RBD up and down configuration [32], S values increase because of the higher MW of the complex, but there is no significant alteration of the distribution of structural conformers. The observed shift corresponds to a molecular mass increment of about 150 kDa, which resembles the mass of C121 IgG (Figure 2b,c and Figure S1).

Antibody sd1.040 binds to a highly conserved part of the SD1 domain, close to the RBD. Its experimentally determined cryptic epitope is only partially exposed on the Spike surface [33]. Opening of the Spike trimer and displacing of the N-terminal domain of adjacent monomers is required to accommodate antibody binding. AUC of the Spike/sd1.040 complex reveals a single, narrow peak distribution at 16.3 S (Figure 2d). Given the above observations, it is tempting to derive that sd1.040 binds to an ‘open’, quaternary conformation of the Spike protein, where the individual monomers are more loosely kept together in comparison to the canonical structure of the trimer.

Regardless of the precise structural features, AUC reveals a process of conformational selection triggered by sd1.040 antibody binding. Spike proteins exist as a mixture of subpopulations whose relative distribution depends on temperature and viral sequence, as seen by AUC. This conformational plasticity transiently exposes the otherwise cryptic sd1.040 epitope, ‘opening’ the trimer to allow its binding. Antibody sd1.040 binds to such an open conformation and then prevents the Spike from ‘closing’ again, resulting in a single, dominant conformer in the Spike/sd1.040 complex. This altered Spike conformation may result in inactivation, explaining the so-far elusive neutralization mechanism of sd1.040 [33]. It is worth noting that it was not possible to obtain a Cryo-EM structure of the complex, perhaps due to such alterations of the trimer structure.

**Table 1:** Sedimentation coefficient values calculated for various Cryo-EM based SARS-CoV-2 Spike D614G trimer structures with WinHydroPro v1.00 [10]. Origin of PDB data: EMDP data source, data deposited by Yang et al. [34].

PDB File	Number of RBD Up	s (S) *
7EB0	1	15
7EB3	1	15
7EB4	2	14.8
7EB5	2	14.7
7EB5MOD	N.A.	12.4

* Sedimentation coefficient calculation was done at 18 °C after verification of effective temperature during the experiment, with corresponding water density and viscosity and applying 433 kDa as molecular mass and 0.73 mL/g partial specific volume.

## 3. Discussion

AUC is a well-established method for protein characterisation, supported by extensive literature [35,36,37] and ISO standards [38,39]. It requires minimal sample preparation and relatively low investment of time and resources.

Here, we show that AUC is not only a powerful tool for quality control of protein products [40,41], but also capable of revealing structural details and conformational variations in solution that are not easily detectable by other experimental or even computational techniques.

AUC analysis of the SARS-CoV-2 Spike protein identifies the presence of structural conformers affected by viral variants, temperature and sample storage conditions. The open structures observed here were found to be more frequently present in samples after prolonged storage at 4 °C—even in S-2P stabilised products [31]. This might explain some inconsistencies and heterogeneous results anecdotally reported in the SARS-CoV-2 field when analysing the Spike and antibodies against it. It highlights the importance of proper quality control for downstream studies, ideally performed with orthogonal techniques including AUC.

We were also able to detect evidence of conformational selection during binding of a neutralizing human antibody, sd1.040, to a cryptic epitope of the Spike protein. These mechanisms are at best difficult to study with other techniques, and underline the novel and largely unexplored value of AUC in the study of (quaternary) conformations and molecular rearrangement.

## 4. Methods

### 4.1. Protein Expression and Purification

S-2P mutant SARS-CoV-2 recombinant Spike proteins were synthesised by the group of Prof. Luca Varani (Bellinzona, IRB, CH), as previously described [16,33]. A codon-optimized gene encoding residues 1 to 1208 of the SARS-CoV-2 S ectodomain (GenBank: MN908947) was synthesized and cloned into the mammalian expression vector pcDNA3.1(+) by GenScript; the sequence contains proline substitutions at residues 986 and 987 (S-2P), a “GSAS” substitution at the furin cleavage site (residues 682 to 685), a C-terminal T4 fibritin trimerization motif, and a C-terminal octa-histidine tag. SARS-CoV-2 S ectodomains corresponding to the SARS-CoV-2 VOCs were based on the following: Delta, GenBank: QWK65230.1; Omicron BA.1 GenBank: UFO69279.1.

All the Spike proteins were produced by transient polyethylenimine (PEI) transfection in Expi293F cells (Thermo Fisher Scientific, Monza, Italy), purified from the cell supernatants six days after transfection by HiTrap Chelating HP (Cytiva, MA, USA).

Antibodies (Abs) were synthesised by Prof. Luca Varani and Prof. Davide Robbiani groups (Bellinzona, IRB, CH [32,33]). Plasmids containing the coding sequence for the production of the monoclonal antibodies C121 and sd1.040 were prepared as previously described [33,42]. These monoclonal antibodies were produced by transient PEI transfection in Expi293F cells (ThermoFisher, Monza, Italy), purified from the cell supernatants 6 days after transfection by HiTrap Protein A HP (Cytiva, Marlborough, MA, USA) and HiLoad Superdex 200 16/60 column (Cytiva, Marlborough, MA, USA).

All proteins underwent quality control and biophysical characterization to ensure functionality, stability, lack of aggregation and batch-to-batch reproducibility. Proteins were stored at −80 °C.

### 4.2. SDS-PAGE

To evaluate protein size and amount electrophoresis analysis was performed using the High Sensitivity Protein 250 kit assay (Cat 5067-1575, Agilent Technology, Santa Clara, CA, USA) on the 2100 Bioanalyzer following manufacturer’s instructions. Briefly, each sample was equilibrated using the Standard labelling buffer and labelled with the fluorescent dye. Samples were run both under reducing and non- reducing conditions. In case of reducing conditions, 1M DTT (3.5 vol%) was added to the sample before boiling. Samples were diluted 200 times in milli Q-water and denatured at 95 °C for 5 min. The samples were cooled and loaded on the microfluidic chip for the electrophoresis. All reagents and instruments were from Agilent Technology ( Santa Clara, CA, USA).

### 4.3. AUC

To analyse the sedimentation coefficient distribution of the SARS-CoV-2 Spike protein and the binding of the different antibodies to the trimer, sedimentation velocity type experiments were performed using a Beckman Coulter Proteomlab XL-I analytical ultracentrifuge (Brea, CA, USA) equipped with an 8 hole rotor. Samples were incubated 30 min at room temperature or 2 days at 4 °C, followed by 4 h at 37 °C and diluted in PBS (Gibco, NY, USA) to a final volume of 380 µL with nominal Spike trimer concentration of 0.53 µM and nominal concentration of 1.73 µM in the case of antibodies. Mixtures were then incubated for 30 min at room temperature before the loading in a double sector cells with sapphire windows. PBS was used as reference at a volume of 390 µL. Interference optics was applied to register the change in refractive index difference at 40,000 rpm rotational speed at a nominal temperature of 20 °C. Experiments were repeated twice using the same batch of proteins stored under the exactly same conditions.

### 4.4. Calculations and Model Fits

Sedimentation coefficient (s) distributions of the molecules were determined using the Continuous c(s) model of Sedfit [43] choosing a range of 0–30 Svedberg for the fit, a linear grid with resolution of 400 and a fixed frictional coefficient ratio (f/f0) value of 1.6. The fit and the molecular mass estimation were done using a density and viscosity of 1 g/mL and 0.01 mPa·s for the solvent and a partial specific volume of 0.73 mL/g for protein molecules. Origin v16 was used for deconvolution of the s distributions. WinHydropro v1.00 [10] was utilised to calculate the sedimentation speed of various conformation variants by using deposited PDB files as input data from the RCSB protein databank. UCSF Chimera [44] was used to further modify the deposited structure of the “unfolded” trimer conformation and to generate a PDB file of the open, slowly sedimenting form of the trimer.

## Data Availability

The data presented in this study are available on request from the corresponding author.

## Supplementary Materials

The following are available online at https://www.mdpi.com/article/10.3390/ijms241914875/s1.
