# Supplementary material for: Analytical Ultracentrifugation Detects Quaternary Rearrangements and Antibody-Induced Conformational Selection of the SARS-CoV-2 Spike Trimer
Source: Int J Mol Sci. 2023 Oct 3;24(19):14875. doi: 10.3390/ijms241914875 (PMC10573103; doi:10.3390/ijms241914875)
Supplement: Supplementary file 1 [file ijms-24-14875-s001.zip › ijms-2585616-supplementary.pdf]

Supplementary Materials

Analytical Ultracentrifugation Detects Quaternary Rearrangements and Antibody-Induced Conformational Selection of the SARS-CoV-2 Spike Trimer

Giuditta Guerrini [1], Dora Mehn [1], Francesco Fumagalli [1], Sabrina Gioria [1], Mattia Pedotti [2], Luca Simonelli [2], Filippo Bianchini [2], Davide F. Robbiani [2], Luca Varani [2,*] and Luigi Calzolai [1,*]

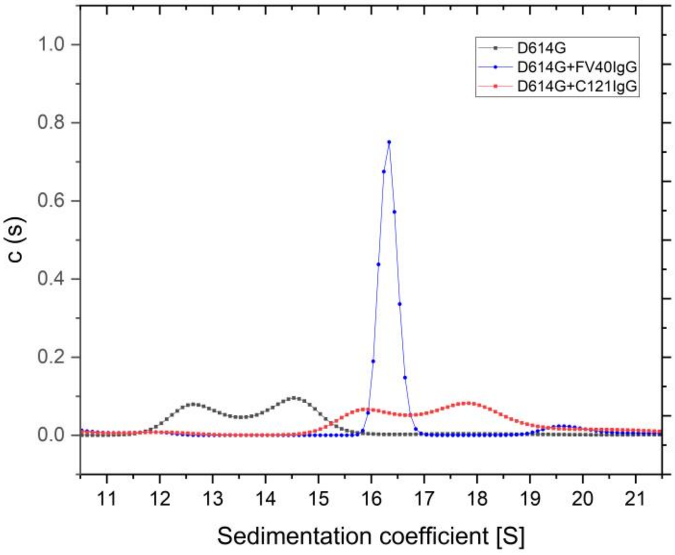

Figure S1. AUC sedimentation coefficient distribution of D614G Spike protein. Effects of antibody binding on the different quaternary conformations. Black: protein alone; red: spike protein bound to C121 IgG; blue: spike protein bound to sd1.040 IgG.
